# Periodic Phase Slips and Frequency Comb Generation at Tunable Microwave Frequencies in Superconducting Diabolo Structures

**DOI:** 10.1021/acsnanoscienceau.5c00056

**Published:** 2025-06-22

**Authors:** Axel J. M. Deenen, Dirk Grundler

**Affiliations:** † Laboratory of Nanoscale Magnetic Materials and Magnonics, Institute of Materials (IMX), School of Engineering, École Polytechnique Fédérale de Lausanne (EPFL), Lausanne 1015, Vaud. Switzerland; ‡ Institute of Electrical and Micro Engineering, School of Engineering, École Polytechnique Fédérale de Lausanne (EPFL), Lausanne 1015, Vaud. Switzerland

**Keywords:** superconductors, three-dimensional nanodevice, frequency comb, nonequilibrium superconductivity, phase slip, time-dependent Ginzburg−Landau, vortex

## Abstract

Superconductors are characterized by macroscopic phase coherence and have enabled cryogenic electronics and quantum technologies. Recent advances in 3D nanofabrication now offer possibilities for tuning functional properties relevant for on-chip 3D integration of superconductors. However, nonequilibrium phenomena in 3D nanostructures exposed to transport currents remain largeley unexplored. Here, we employ numerical simulations to investigate phase slips—discrete 2π jumps in the phase of the superconducting order parameter—in a tubular Nb superconductor with a central constriction, which is subjected to both direct current (DC) and alternating current (AC) transport currents. We find that under DC drive, the system stabilizes periodic phase slips, resulting in GHz voltage oscillations. Introducing an additional AC frequency modulation generates microwave frequency combs which depend characteristically on the interaction between moving vortices and phase slips. Our findings open avenues for developing on-chip frequency comb generators in 3D cryoelectronics.

## Graphical abstract

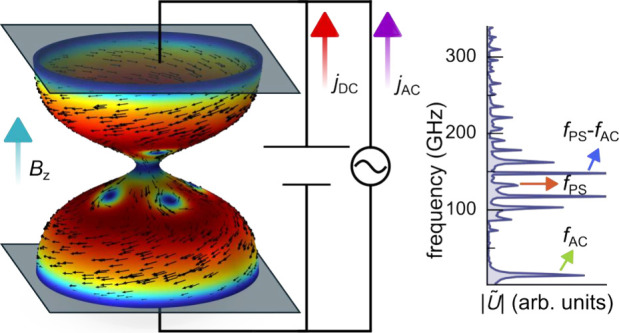

## Introduction

Superconductors are at the forefront of nanoscience and quantum technology, underpinning innovations in qubits, ultrasensitive sensors, and parametric amplifiers. Central to many of these technologies is the Josephson junction, typically formed by two superconductors (S) separated by a thin insulating barrier (I). Recently, generation of frequency combs using Josephson junctions and a superconducting resonator was demonstrated.^1^

SIS technology continues to underpin superconducting electronics. Still, its multilayered structure incorporating an ultrathin tunnelling barrier limits geometric flexibility, its orientation on a chip and exploitation on 3D curved surfaces. In particular, for three-dimensional (3D) circuits containing several functional levels, vertical interconnects, so-called vias, are needed. An alternative and complementary approach to Josephson effects which is compatible with realizing in-plane and out-of-plane interconnects in arbitrary orientations involves the use of weak links.^2,3^ A typical example consists of two superconducting banks connected by a superconducting constriction, often referred to as a micro- or nanobridge.^4^ Typically, when the width and length of these constrictions are smaller than the coherence length (ξ), Josephson effects can be observed.

Additionally, a current-driven superconductor with a weak link can exhibit a resistive state characterized by periodic suppression of the order parameter magnitude and 2π phase slips.^5,6^ Phase slips manifest as phase-slip centers^7^ in quasi-one-dimensional systems or as phase-slip lines^8^ in wider films and induce quantum interference effects similar to Josephson junctions.^9^ They have been proposed for use in flux qubits^10^ and optimizing transmon qubits.^11^ Recent advances in nanofabrication have enabled the realization of 3D superconducting architectures with nanoscale precision,^12–15^ and have led to the realization of free-standing nanowires with diameters down to less than 40 nm,^16^ making controllable constriction-based phase slips such as nanobridges^17^ especially promising for high-density 3D sensing and computing architectures.

However, while phase slip phenomena in one dimension are relatively well understood,^18,19^ analytical descriptions in two and three dimensions remain challenging due to the complexity of the governing equations. In particular, the interplay between topological defects, such as vortices and phase slips,^13,20^ in 3D nanostructures is not fully understood.

In this work, we conduct full 3D time-dependent Ginzburg–Landau simulations to investigate phase slip phenomena in Nb diabolo structures—hollow tubes featuring a central constriction (Figure 1a)—under both DC and combined DC+AC current drives. We demonstrate that the intrinsic frequency of phase slip oscillations, given by the Josephson relation,^21^ can be harnessed to generate microwave frequency combs, potentially advancing high-frequency quantum applications. Furthermore, we demonstrate the coexistence of phase slips with vortices and show that at sufficiently high currents, vortices are absorbed by the phase slip region, offering means of controlling topological defects and microwave spectra by transport currents in superconducting systems.

**Figure 1:**
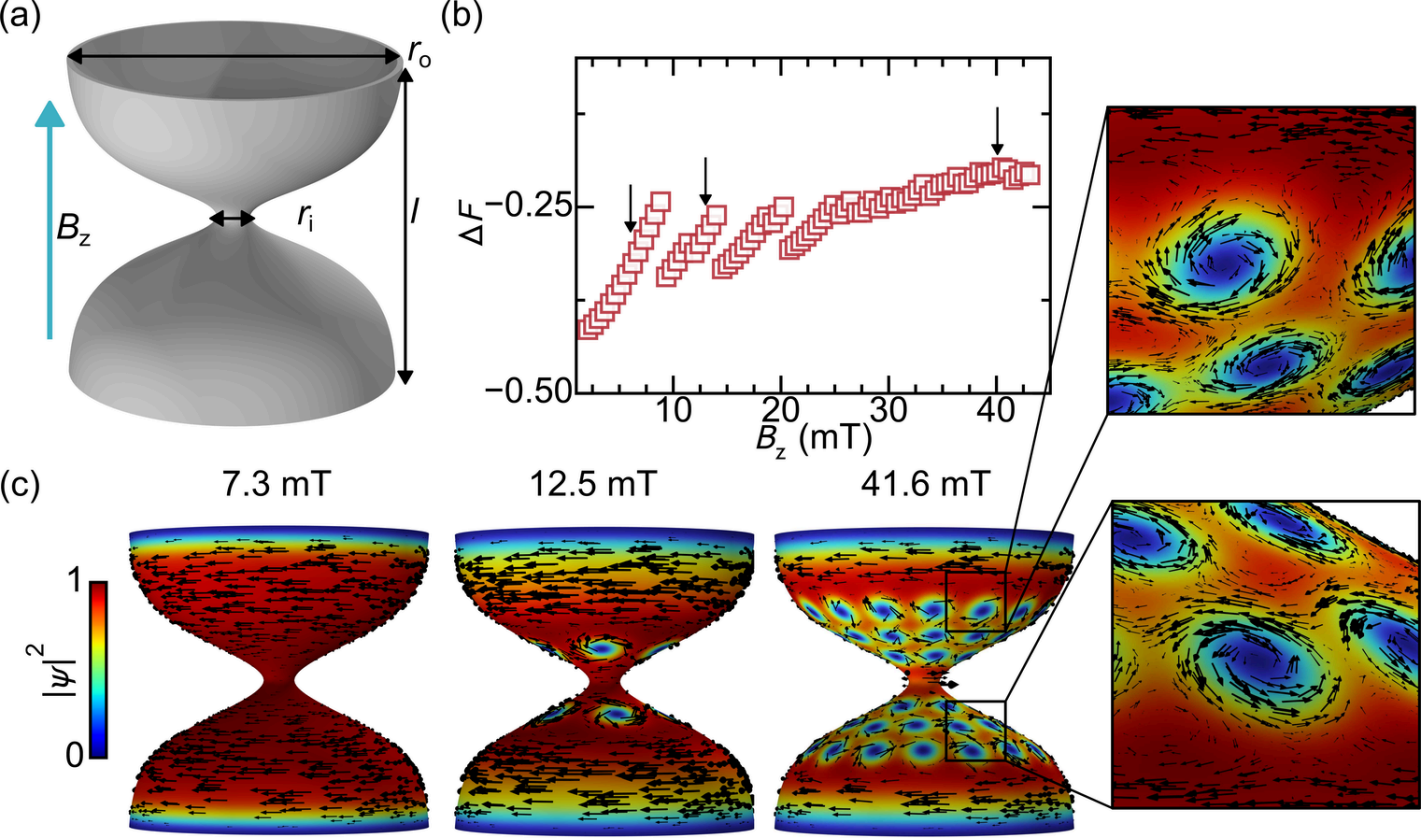
States of the diabolo under constant applied axial field. (a) Geometry of the diabolo highlighting the inner radius *r*_*i*_, the outer radius *r*_*o*_, the length *l* and the applied field *B*_*z*_. (b) The computed free energy difference Δ*F* between the superconducting state and the normal state as a function of field. (c) The spatial distribution of the order parameter magnitude at several fields indicated by arrows in (b).

## Results and Discussion

We have numerically simulated several tubular structures of different geometrical parameters and report here on a superconducting Nb diabolo structure (Figure 1a). It consists of an Nb shell with a thickness of *d* = 27 nm and length *l* = 1.64 μm. The radius varies from *r*_0_ = 890 nm at the ends to *r*_i_ = 58 nm at the central constriction. Throughout this work, we consider a fixed temperature *T* = 0.95*T*_c_, with *T*_c_ the critical temperature. The penetration depth and coherence length are taken as λ = 273 nm and ξ = 58 nm, respectively, giving a Ginzburg–Landau parameter κ = λ/ξ = 4.7.^22^ The normal conductivity σ = 16 (μΩ m)^−1^. Since *d* ≪ λ, self-field effects are neglected. To study the dynamic response, we numerically solve the time-dependent Ginzburg–Landau (TDGL) equations using a 3D finite-element formulation^23^ implemented in COMSOL Multiphysics.^24,25^ We adopt the Coulomb gauge,^26^ imposing ∇·**A** = 0 on the vector potential **A**.

Within the Ginzburg–Landau framework, the dimensionless free energy difference between the normal and superconducting states, expressed in terms of the complex order parameter ψ, is given by

1 $$\Delta F=-{\left|\psi \right|}^{2}+\frac{1}{2}{\left|\psi \right|}^{4}+{\left|\left(\frac{1}{\kappa }\nabla -iA\right)\psi \right|}^{2}+{\left|\nabla \times A-H\right|}^{2}$$

Here, lengths are normalized to λ, and time to ξ^2^/*D*, with *D* being the diffusion coefficient. The last term in eq 1 is negligible in the limit of small self-fields. The evolution of ψ is described by the TDGL equation:^23,27^

2 $$\left(\frac{\partial }{\partial t}+i\kappa \phi \right)\psi ={\left(\frac{1}{\kappa }\nabla -iA\right)}^{2}\psi +\left(1-{\left|\psi \right|}^{2}\right)\psi$$

supplemented by the Poisson equation for the scalar potential ϕ to ensure current conservation:

3 $$\sigma \nabla^{2}\phi =-\nabla \cdot \left(\frac{i}{2\kappa }\left(\psi^{*}\nabla \psi -\psi \nabla \psi^{*}\right)+{\left|\psi \right|}^{2}A\right)$$

where σ is the normal conductivity. Boundary conditions are applied as follows:

- At the superconductor/vacuum (SV) interface: ∇ψ·*n̂* = 0, ∇ϕ·*n̂* = 0, and A·*n̂* = 0.
- At the superconductor/metal (SN) interface: ψ = 0 and $\nabla \phi \cdot \mathrm{n̂}=-j_{tr}/\sigma$ .

with *j*_tr_ the applied transport current. Additionally, to ensure uniqueness of ϕ,^26^ we impose the condition:

4 $$\int_{\Omega }\phi \;d\Omega =0$$

where Ω denotes the simulated domain.

An external magnetic field *B*_*z*_ is applied along the diabolo’s central axis (*z*-axis). The vector potential **A** satisfies the gauge condition and boundary conditions and is expressed as

5 $$A=A_{ext}+\nabla \chi$$

where $A_{ext}=\frac{1}{2}B_{z}xŷ-\frac{1}{2}B_{z}y\mathrm{x̂}$ corresponds to the symmetric gauge, and χ is a gauge function solving the Laplace equation:

6 Δχ=0

subject to the boundary condition:

7 $$\nabla \chi \cdot \mathrm{n̂}=-A_{ext}\cdot \mathrm{n̂}$$

on Γ, the superconductor boundary. The scalar potential is decomposed^28,29^ as ϕ = ϕ_1_ + ϕ_2_, satisfying:

8 $$\nabla^{2}\phi_{1}=0,\;\nabla \phi_{1}\cdot \mathrm{n̂}=-j_{tr}/\sigma ,on\;\Gamma$$

and

9 $$\nabla^{2}\phi_{2}=\frac{1}{\sigma }\nabla \cdot {\mathrm{j⃗}}_{sc},\;\nabla \phi_{2}\cdot \mathrm{n̂}=0,on\;\Gamma$$

The external field is incrementally swept^29^ in steps of *B*_*z*_ = 0.51 mT, with the system relaxed at each step for 700.9 ps or until the convergence criterion is met:

10 $$\frac{1}{\Omega }\int_{\Omega }dx\frac{\partial {\left|\psi \right|}^{2}}{\partial t}<10^{-9}$$

Figure 1b presents the free energy difference Δ*F* as a function of the applied field. The energy generally increases with increasing field, except at specific values where discrete jumps to lower energy states occur. These jumps correspond to vortex nucleation events.

Figure 1c illustrates the order parameter distribution below and above the first energy jump. At *B*_*z*_ = 7.3 mT, the system remains in a pure superconducting state, exhibiting azimuthal screening currents. At *B*_*z*_ = 12.5 mT, vortices have nucleated above and below the constriction, forming rows with opposite vorticity and phase windings, behaving as vortices and antivortices. This configuration arises from the structure’s curvature, leading to an inhomogeneous projection of the applied field on the surface normal.

We next investigate the dynamics under a DC transport current. Current is injected at the edges via appropriate boundary conditions (Figure 2a), with the applied field set to *B*_*z*_ = 7.3 mT, below the vortex nucleation threshold. Above a critical current, the system enters a dynamic equilibrium characterized by periodic suppression of the order parameter and GHz voltage oscillations, marking the phase slip regime.

**Figure 2:**
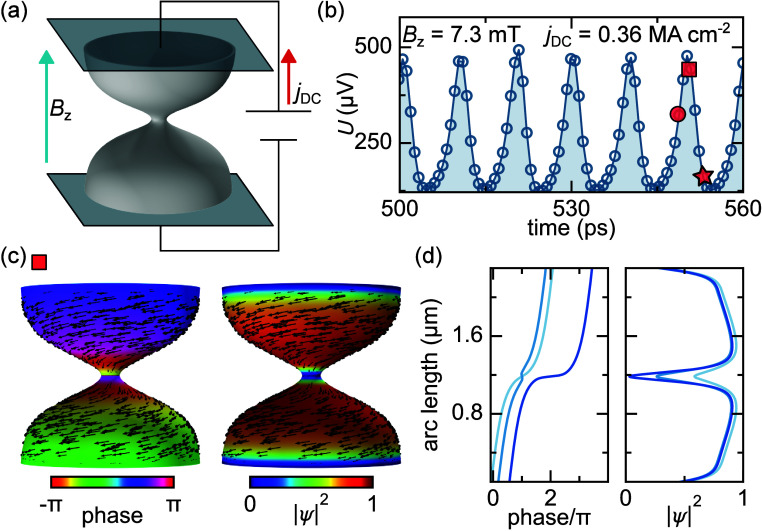
Diabolo under applied DC transport current below the lower critical field. (a) Schematic of how the leads are applied. (b) Stable voltage oscillations under DC current. Three points during the oscillations highlighted with red symbols are further analyzed. (c) The spatial distribution of the phase and magnitude of the order parameter during a phase slip at the peak of the voltage. (d) Lineplots of the unwinded phase and order parameter magnitude highlight the suppression of the order parameter and a subsequent jump in phase.

Figure 2b presents the time-dependent voltage *U* between the top and bottom leads as a function of time at an applied current of *j*_DC_ = 0.36 MA cm^–2^. The voltage *U* was determined as the difference in the total scalar potential ϕ, averaged over the top and bottom leads of the diabolo:

$$U=\frac{1}{\Gamma ,top}\int_{\Gamma ,top}dl\phi -\frac{1}{\Gamma ,bot}\int_{\Gamma ,bot}dl\phi$$

where the integration is done over the top and bottom perimeter Γ,top and Γ,bot, respectively. To understand this regime, we analyze the spatial distribution of the order parameter’s phase and magnitude (Figure 2c). At the voltage peak (indicated by a square symbol), the order parameter is fully suppressed at the constriction. T The phase profile is rotationally symmetric and varies along the arc length of the diabolo. Figure 2d illustrates the phase variation (left) and order parameter (right) along the arc length for three different points in time. The phase undergoes a 2π jump at the constriction during each phase slip. As described by Michotte et al.,^30^ the periodic phase slip state is stable when the relaxation time of the order parameter phase is shorter than that of the magnitude, i.e., τ_ϕ_ < τ_|ψ|_.

The phase slip regime persists in the presence of vortices. Figure 3 presents a DC current sweep performed at an applied field of 12.5 mT, where the diabolo hosts two vortex rows (see Figure 1c). At low DC currents, vortices and antivortices begin to move under the influence of the Lorentz force. Due to the opposite circulation of their screening currents, their motion under the transport current occurs in opposite directions—either clockwise or counterclockwise. This motion results in a finite, time-constant voltage *U*. Due to the tubular diabolo geometry, the vortex motion in the absence of pinning is uninterrupted, avoiding the nucleation and annihilation processes at the boundary that would lead to voltage spikes in a planar (conventional) constriction. As the DC current increases, the diabolo passes into the phase slip regime. Notably, the frequency of phase slips scales with the DC current (Figure 3b). This frequency is determined by the Josephson relation, which relates the phase slip frequency *f*_ps_ to the average voltage drop over the phase slip $\mathrm{U̅}=\frac{h}{2e}f_{ps}$ .^21^ Additionally, higher-order harmonics appear in the voltage spectrum, which are attributed to the system’s intrinsic nonlinearity. The time-averaged voltage ⟨*U*⟩ and the corresponding differential voltage *d*⟨*U*⟩/*dj* are shown in Figure 3c and 3d, respectively. Both reveal distinct jumps at *j*_DC_ = 0.6 MA cm^–2^ and *j*_DC_ = 0.66 MA cm^–2^. During these transitions, the vortex and antivortex rows are absorbed into the constriction by the phase slip. Depending on *j*_DC_, this process leads to periodic vortex–antivortex pair nucleation and annihilation within the constriction (Figure 3e).

**Figure 3:**
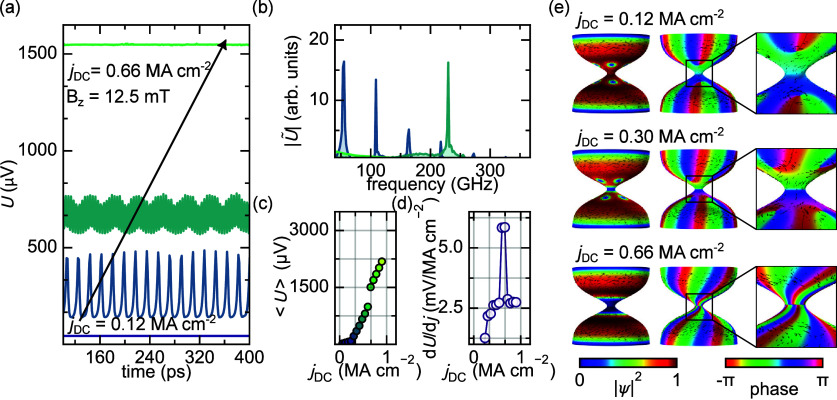
DC current sweep in the mixed state. (a) Voltage, time diagram at constant applied field and various applied DC transport currents. (b) The Fourier transformed signals shown in (a). (c) Time averaged voltage as a function of DC current and (d) the differential time averaged voltage as a function of DC current, i.e. differential resistance. (e) Spatial distribution of the order parameter magnitude and phase highlighting the various topologically distinct states at the constriction.

The phase slips and their DC-current-controlled GHz oscillations open up an intriguing functionality. This is demonstrated by introducing an additional AC current modulation *j*_AC_ = *j*_0_ sin­(2*πf*_AC_ *t*) to the DC current *j*_DC_ when the sample is in the phase slip regime (Figure 4a). Here, *j*_0_ is the AC amplitude, which is fixed at *j*_0_ = 0.036 MA cm^–2^, and *f*_AC_ = 15 GHz is the modulation frequency. We start from an initial state with *B*_*z*_ = 12.5 mT and *j*_DC_ = 0.36 MA cm^–2^, ensuring a stable phase slip regime (Figure 3). The AC amplitude *j*_0_ is gradually ramped^29^ from 0 to 0.036 MA cm^–2^. Subsequently, the AC amplitude and frequency are held constant while sweeping over the DC amplitude. The resulting *j*_DC_-dependent spectra (Figure 4b) possess several noteworthy features. Instead of a single fundamental peak and its harmonics, the introduction of the AC signal leads to a range of harmonic peaks. This frequency mixing arises from the nonlinearity of the superconducting condensate, resulting in frequencies of the form *f* = *nf*_ps_ + *mf*_AC_, where *n* and *m* are integers, and *f*_ps_ is the fundamental phase slip frequency driven by the DC current. This mechanism realizes a frequency comb with *n* = 1 consisting of hierarchies of equidistant frequency peaks for a fixed *j*_DC_. Additionally, a second set of branches with a steeper slope is observed for small *j*_DC_ which corresponds to the *n* = 2 harmonics. Further simulations indicate that the width of the frequency comb scales with the AC amplitude, while the comb spacing is determined by the modulation frequency *f*_AC_ (Supporting Information). A distinct jump in the spectrum is observed, which is reflected in both the time-averaged voltage and the differential voltage (Figure 4c). This discontinuity corresponds to the entrance of a single vortex and antivortex pair into the constriction.

**Figure 4:**
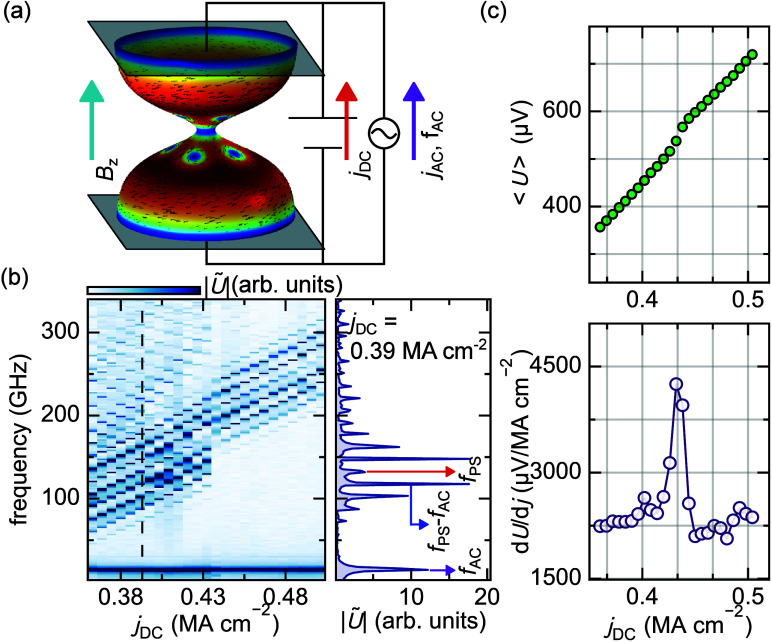
DC current sweep of the diabolo under constant applied field and AC current. (a) schematic of the simulation, highlighting that the injected DC transport current is now modulated with an AC current at constant amplitude and frequency. (b) Resulting Fourier transformed voltage |Ũ| as a function of DC current amplitude. The spectrum shows nonlinear frequency mixing of the phase slip frequency *f*_ps_ with the ac current frequency *f*_*AC*_. The right side shows a linecut of the spectrum taken at *j*_*DC*_ = 0.39 MA cm^–2^ (dashed line). (c) Time averaged voltage (top) and differential time averaged voltage (bottom) as a function of DC current.

Prior to the jump, the phase evolution resembles that shown in Figure 2, where the phase distribution within the constriction exhibits rotational symmetry. At the onset of the jump, an isolated vortex first enters the constriction and moves at high velocities. Subsequently, at *t* ≈ 420 ps, an antivortex enters the constriction. This leads to the disappearance of the higher-order frequency branches in the spectrum. At the higher DC currents shown in Figure 4b, no additional vortices are observed to enter the constriction. Moreover, upon subsequently reducing the DC current, the system does not exhibit a reappearance of the jump, featuring the presence of hysteresis in the current–voltage characteristic.

The phase slips observed in this study within a multiply connected diabolo structure—characterized by a hollow tube—exhibit distinct behavior compared to those in a 1D wire. At a sufficiently high current, a transition occurs wherein one or more vortices enter the constriction (middle to bottom panel of Figure 3e), fundamentally altering the nature of the phase slip. Initially, the phase slip exhibits a 1D character with phase variations only longitudinal to the current. However, after the transition, the phase slip becomes two-dimensional in nature and is characterized by both transverse and longitudinal phase variations due to the periodic annihilation of vortex–antivortex pairs (bottom panel Figure 3e). For thin films or hollow cylinders, such transitions between 1D and 2D phase slips take place only as a function of width or radius.^31^ In a diabolo nanostructure, however, this transition can take place as a function of current since both vortices and phase slips are stabilized. This event is marked by a peak in differential resistance and a notable change in the voltage spectrum under AC modulation. Specifically, the absorption of one or more (anti)­vortices induces discrete jumps in the spectrum and suppresses higher-frequency modes.

The superconducting diabolo considered in this work was free of defects. In a real device, vortex pinning, thickness variations, and disorder might be present. Here we briefly discuss the impact of these factors and a possible self-heating effect. We have shown that at low enough fields (Figure 1), no vortices are present in the system and a pure phase slip state is entered (Figure 1). For the resulting frequency comb, vortices and corresponding pinning centers [^32^ do not play a role. Film thickness variations are negligible as long as the thickness remains below the coherence length and the diabolo shell can be treated as a 2D sheet.^22^ *T*_*C*_ inhomogeneity and disorder could lead to a local suppression of the order parameter and create “weak spots” where the critical current for phase slips is lowered.^5^ However, by design the current density is maximum in the constriction of the diabolo geometry. The widened ends of the diabolo lead to a low current density outside of the constriction, ensuring that the phase slip is localized at the center. The functionality of the proposed device does not require the presence of vortices, hence self-heating effects due to vortices are not detrimental in principle. For the situation where both vortices and phase slips are present such as in Figures 3 and 4, efficient heat removal could be realized by either coating the superconductor with an effective heat conduit, or by depositing the superconductor on such a conduit. Additionally, one can connect the system in parallel with a shunt.

We have investigated the phase slips in the tubular 3D nanostructures by employing the TDGL equations, which are valid for gapless superconductors near the critical temperature.^27^ Previous studies have shown that the onset current for phase slips depends on the relaxation times of both the order parameter’s magnitude and phase.^30,33^ The magnitude relaxation is influenced by the finite inelastic scattering time, which could be incorporated using the generalized TDGL formalism.^34^ Although this refinement might quantitatively affect the results, the standard TDGL framework has been extensively applied to study phase slips in one- and two-dimensional systems and reliably captured the essential behavior qualitatively.^8,35,36^

## Conclusion

We have numerically investigated the static and dynamic properties of three-dimensional Nb diabolo structures, where the constriction radius is comparable to the coherence length (ξ). Our primary finding is that a DC-driven diabolo functions as a DC-to-AC converter and, when exposed to an additional AC signal, acts as a frequency comb generator in the microwave regime.

The curved geometry of the diabolo structure supports the formation of both vortices and antivortices in response to a homogeneous magnetic field. At sufficiently high DC currents, a stable phase-slip regime emerges, characterized by the periodic suppression of the order parameter within the constriction. This phase-slip state can coexist with the vortex lattice. As the current increases, the phase-slip region expands and eventually absorbs the vortex rows, resulting in periodic vortex–antivortex annihilation events, which manifest as a distinct peak in the differential voltage. Furthermore, introducing microwave-frequency modulation to the transport current induces frequency mixing and generates sidebands. Notably, when the phase-slip region has absorbed vortices, higher-order frequency modes are suppressed.

Our findings open possibilities for developing 3D integrated, on-chip frequency comb generators by conformal coating of a pillar-like nanotemplate with a single superconducting shell. Conformal coating of 3D superconductors can be achieved via several methods, for instance via atomic layer deposition (ALD).^37^ ALD of superconductors is an established process and has recently received renewed interest due to the prospects of 3D superconducting applications.^38^ To generate complex 3D structures, ALD can be combined with a 3D polymeric template written by multiphoton lithography, as has been demonstrated recently for ferromagnets.^39^ The diabolo structure described in this manuscript uses only a single superconducting material, Nb, and could also be realized by using a direct write method based on a focused ion beam, since the smallest feature sizes considered are ∼ 30 nm, comparable with the state-of-the-art.^16,40^ Our design offers a promising advancement for quantum nanotechnologies and high-frequency applications.

## Supplementary Material

















## Notes

Additional details on numerical procedure, simulations on wider constrictions, frequency comb dependence on AC and DC current strength (PDF)
Nulceation of vortices during a field sweep (AVI)
Magnitude of the order parameter at *j*_DC_ = 0.12 MA cm^−2^ (AVI)
Phase of the order parameter at *j*_DC_ = 0.12 MA cm^−2^ (AVI)
Magnitude of the order parameter at *j*_DC_ = 0.30 MA cm^−2^ (AVI)
Phase of the order parameter at *j*_DC_ = 0.30 MA cm^−2^ (AVI)
Magnitude of the order parameter at *j*_DC_ = 0.66 MA cm^−2^ (AVI)
Phase of the order parameter at *j*_DC_ = 0.66 MA cm^−2^ (AVI)
