# Supplementary material for: Periodic Phase Slips and Frequency Comb Generation at Tunable Microwave Frequencies in Superconducting Diabolo Structures
Source: ACS Nanosci Au. 2025 Jun 22;5(5):362–8. doi: 10.1021/acsnanoscienceau.5c00056 (PMC12532067; doi:10.1021/acsnanoscienceau.5c00056)
Supplement: Supplementary file 1 [file ng5c00056_si_001.pdf]

Supporting Information: Periodic Phase Slips and Frequency Comb Generation at Tunable Microwave Frequencies in Superconducting Diabolo Structures

Axel J. M. Deenen[†] and Dirk Grundler[*,†,‡]

†*Laboratory of Nanoscale Magnetic Materials and Magnonics, Institute of Materials (IMX), School of Engineering, École Polytechnique Fédérale de Lausanne (EPFL), Lausanne, 1015, Vaud, Switzerland*

‡*Institute of Electrical and Micro Engineering, School of Engineering, École Polytechnique Fédérale de Lausanne (EPFL), Lausanne, 1015, Vaud, Switzerland*

E-mail: Dirk.Grundler@epfl.ch

Additional information on numerical procedure

The geometry was meshed using free tetrahedral elements with COMSOL's built-in mesh generator. The maximum element size was set to 0.3ξ, with ξ the coherence length, resulting in a total of 464,898 elements. The thickness of all structures considered in this work was 27 nm. This is much smaller than the assumed coherence length $\xi = 58$ nm. Hence, spatial variations of the order parameter along the thickness can be neglected. The order parameter ψ was decomposed into its real and imaginary components, each solved separately using quadratic Lagrange elements. The scalar potential was computed using linear Lagrange elements. The total system comprised 2,468,930 degrees of freedom and was solved using

the MUMPS solver. Time integration was performed with an implicit second-order Backward Differentiation Formula solver.

Onset of phase slip as a function of constriction radius

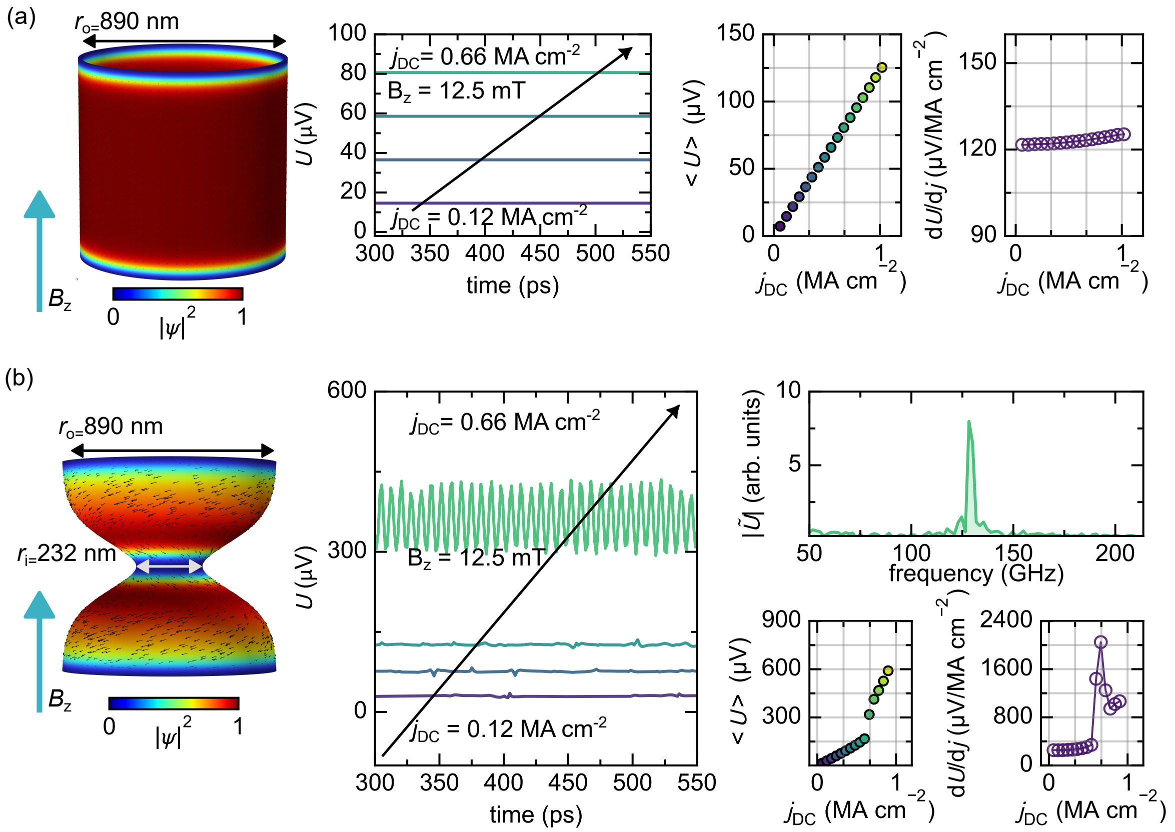

Figure S1: Comparison of the current-voltage characteristic for (a) no constriction and (b) a constriction of inner radius $r_i = 232$ nm. A field of $B_z = 12.5$ mT (identical to the field in Fig. 3 of the main text) was applied along the axis. The range of displayed currents are identical to those of Fig. 3 for the diabolo with constriction radius $r_i = 57$ nm. The needed applied current for the onset of phase slips increases as a function of constriction radius, due to the lowering of current density in the constriction. Additionally, the current range of coexistence of the phase slips and vortices has decreased for the wide diabolo (b) compared to the narrow diabolo (Fig. 3 of the main text), highlighted by the presence of only a single jump in the differential voltage (b) compared to two jumps (Fig. 3).

Frequency comb dependence on AC and DC amplitude

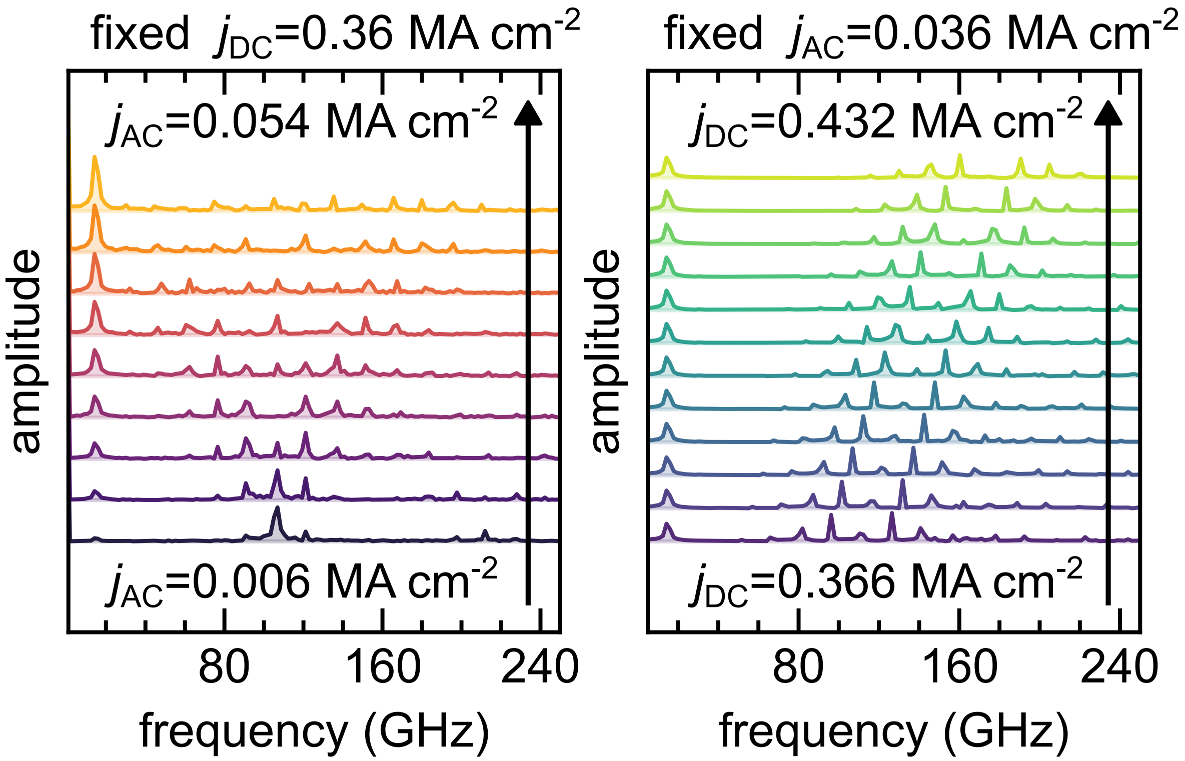

Figure S2: Sweep of the AC modulation amplitude j_{AC} at constant DC current j_{DC} (left) and sweep of the DC current j_{DC} at constant ac amplitude (right) of the diabolo presented in the main text at applied field $B_z = 12.5$ mT. The width of the frequency comb depends on the amplitude of the injected AC signal.
